# Hormone replacement therapy, menopausal age and lifestyle variables are associated with better cognitive performance at follow-up but not cognition over time in older-adult women irrespective of APOE4 carrier status and co-morbidities

**DOI:** 10.3389/frdem.2024.1496051

**Published:** 2025-01-17

**Authors:** Tamlyn J. Watermeyer, Sarah Gregory, Emmi Leetham, Chinedu T. Udeh-Momoh, Graciela Muniz-Terrera

**Affiliations:** ^1^Edinburgh Dementia Prevention, Centre for Clinical Brain Sciences, College of Medicine and Veterinary Sciences, University of Edinburgh, Edinburgh, United Kingdom; ^2^Faculty of Health and Life Sciences, Northumbria University, Newcastle, United Kingdom; ^3^Scottish Brain Sciences, Edinburgh, United Kingdom; ^4^Department of Epidemiology and Prevention, Wake Forest University School of Medicine, Winston-Salem, NC, United States; ^5^Brain and Mind Institute, Aga Khan University, Nairobi, Kenya; ^6^School of Medicine and Population Health, Sheffield Institute for Translational Neuroscience, University of Sheffield, Sheffield, United Kingdom; ^7^Global Brain Health Institute, University of California, San Francisco, San Francisco, CA, United States; ^8^Department of Neurobiology, Care Sciences and Society, Division of Clinical Geriatrics, Karolinska Institutet, Stockholm, Sweden; ^9^Department of Social Medicine, Ohio University Heritage College of Osteopathic Medicine, Athens, OH, United States

**Keywords:** hormone replacement therapy (HRT), menopausal age, cognition, APOE4, cognitive aging, lifestyle factors, postmenopausal women, comorbidities

## Abstract

**Introduction:**

The impact of Hormone Replacement Therapy (HRT) on cognitive function in postmenopausal women remains a topic of considerable debate. Although estrogen's neuroprotective effects suggest potential cognitive benefits, empirical findings are mixed.

**Methods:**

This study uses data from the Cognitive Function and Ageing Study Wales (CFAS Wales) cohort to explore the relationships between HRT use, age at menopause, APOE4 carrier status, lifestyle factors, comorbidities, and cognitive outcomes in older adult women. Two regression models were employed: one analyzing cognitive performance at follow-up and another examining changes in cognitive scores over time.

**Results:**

Results indicate that while age, education, HRT use, age at menopause, alcohol consumption, and diet were associated with cognitive function at a single later time point, only age remained a significant predictor when modeling cognition over time.

**Discussion:**

These findings suggest that while HRT, menopausal age and lifestyle factors may support cognitive stability, they do not necessarily predict cognitive decline in post-menopausal older women. A major limitation of the current work is the lack of detail regarding HRT use, such as formulation, timing and duration; caveats that future studies should address. The study underscores the need for longer follow-up periods, consideration of other female-specific risk factors, and more comprehensive lifestyle and health assessments to clarify the complex interplay between HRT use, reproductive history, lifestyle, comorbidities and cognitive aging in women.

## 1 Introduction

The relationship between Hormone Replacement Therapy (HRT) and cognitive function in postmenopausal women is subject to ongoing investigation and debate (see Mills et al., 2023 for review). The rationale for HRT's potential cognitive benefits lies in the marked decline in estrogen levels during menopause, which is associated with age-related cognitive decline and an increased risk of neurodegenerative diseases, including Alzheimer's disease (AD) (Nerattini et al., 2023). Ovarian hormones are believed to play a crucial role in neurocognitive processes by supporting synaptic plasticity, neuroprotection, and cerebral blood flow regulation (Jett et al., 2022). Consequently, the potential for HRT to mitigate cognitive decline has generated significant interest, particularly in light of the higher prevalence of AD in women compared to men (Buckley et al., 2019; Mielke, 2018; Riedel et al., 2016), understanding the role of HRT in cognitive aging is of significant clinical importance (Ferretti et al., 2018) and relevant for secondary prevention strategies that target sex-specific risk factors (Udeh-Momoh and Watermeyer, 2021).

Despite these theoretical benefits, empirical findings on HRT's impact on cognition have been inconsistent (see Nerattini et al., 2023 for systematic review). Observational studies have frequently reported a positive association between HRT use and cognitive performance on global as well as domain scores, such as memory, processing speed and executive functions (Coughlan et al., 2022; Saleh et al., 2023; Yaffe et al., 1998). Similarly, HRT use has been associated with greater brain volumes in key AD-relevant regions, such as the entorhinal cortex, the hippocampus and prefrontal cortex (Saleh et al., 2023; Coughlan et al., 2023) and lower levels of AD-relevant biomarkers, such as phosphorylated tau (p-tau) and total tau (t-tau) (Lee et al., 2024). The influence of APOE4 carriership is controversial, with evidence suggesting that these effects are relevant only or more for women carrying the APOE4 allele (Saleh et al., 2023; Depypere et al., 2023), while other evidence suggests that only non-carriers derive cognitive benefits from HRT (Burkhardt et al., 2004). Still other evidence indicates no relevance of APOE4 status to female-specific factors and cognition (Lindseth et al., 2023). The influence of menopausal age alongside HRT use may also be relevant, with some studies noting that later menopausal age for HRT users is associated with better cognitive performance and reduced dementia risk in later life (Park et al., 2024), suggesting that longer exposure to endogenous and exogenous estrogen may be neuroprotective (Lee et al., 2024).

Conversely, randomized controlled trials (RCTs) have generally produced less favorable results in terms of cognitive benefits from HRT use. The Women's Health Initiative Memory Study (WHIMS, Shumaker et al., 1998), found that HRT was associated with an increased risk of dementia and cognitive decline when initiated in women aged 65 and older (Coker et al., 2010). This study significantly influenced clinical guidelines, leading to a more cautious approach to HRT use in older women. Additionally, the Kronos Early Estrogen Prevention Study (KEEPS) failed to demonstrate significant cognitive benefits of HRT in newly menopausal women (Gleason et al., 2015). More recently, a prospective trial of 6-months estrogen therapy in HRT-naïve cognitively healthy and younger menopausal women showed longitudinal changes in AB/p-tau ratio scores for women within the HRT arm; these effects strongest for APOE4 carriers (Depypere et al., 2023), indicating, for the first time, potential positive effects of HRT on biomarkers relating to AD pathophysiology. However, cognition was not assessed in this study due to the relatively short follow-up period.

The discordance between observational studies and RCTs could stem from differences in study populations, HRT formulations (i.e., estrogen-only, progesterone-only or combined preparations), and, crucially, the timing of HRT initiation. The “critical window hypothesis” posits that HRT is most effective when initiated near the onset of menopause, during a period when the brain may be more responsive to estrogen (Maki and Sundermann, 2009; Udeh-Momoh and Watermeyer, 2021). Similarly, the importance of considering cardiovascular health, metabolic status, and lifestyle factors when evaluating the cognitive effects of HRT has also been highlighted (Lee et al., 2024; Espeland et al., 2015; Liao et al., 2023; Rocca et al., 2024), despite several comorbidities representing exclusion criteria for HRT RCTs. RCT studies of the Mediterranean diet suggest significant improvements in cognitive domain composites and evidence for modulation of AD-relevant genotypes in dementia risk (Radd-Vagenas et al., 2018). Separately, adherence to this diet, which prescribes a high-intake of vegetables, nuts and their oils, has shown benefits to menopausal women's health, through weight and cardiovascular management (Gonçalves et al., 2024; Gregory et al., 2023b). Such benefits might occur through the estrogenic properties of recommended foods associated with the diet (Rispo et al., 2024).

While HRT remains a commonly prescribed treatment for menopausal symptoms, its impact on cognitive health remains controversial. Based on the existing body of literature, this study seeks to clarify the complex relationship between HRT and cognitive function in postmenopausal women, considering the potential influence of key factors such as age at menopause, APOE4 carrier status, lifestyle factors, and comorbidities. By leveraging data from the Cognitive Function and Ageing Study Wales (CFAS Wales) cohort of older-adults to explore whether HRT use is associated with better cognitive outcomes in older women, and how these outcomes are potentially moderated or mediated by such variables.

## 2 Methods

### 2.1 Study design and participants

This study uses data from the Cognitive Function and Ageing Study Wales (CFAS Wales), a population-based longitudinal cohort study focused on individuals aged 65 and older residing in Wales. The study investigates various risk factors and health outcomes among this population, and previously has explored the influence of socio-economic factors, bilingualism, cognitive reserve and lifestyle on cognitive status in later life across the entire sample (men and women) (Gamble et al., 2022; The Medical Research Council Cognitive Function and Ageing Study, 1998; Clare et al., 2017; Jia et al., 2021). It was conducted in two distinct geographical areas: a rural region (Gwynedd and Ynys Môn) and an urban area (Neath Port Talbot). All participants were consented to the study using informed consent procedures. The study included two waves of data collection: baseline data were gathered between 2011 and 2014, with a follow-up conducted 2 years later, from 2013 to 2016. The study received ethical approval from the NHS North Wales—West Research Ethics Committee (REC Ref No: 10/Wno01/37; IRAS Project No: 40092). The original study inclusion criteria comprised being born before 1946 and English or Welsh proficiency. For the purposes of our study, we included only female participants without evidence of cognitive impairment at the baseline assessment, as indicated by a Mini-Mental State Examination (MMSE, Folstein et al., 1975) score of 25 or higher. The study sample included 629 individuals who met the inclusion criteria.

### 2.2 Variables of interest

#### 2.2.1 Demographic, reproductive history and APOE4 carrier status

Age was recorded at baseline; education was quantified as number of years of formal education. At the baseline visit, participants were asked the year of their last period. From this year, 1 year was added to represent their menopausal age, in keeping with the STRAW criteria (Harlow et al., 2012). Whether menopause was natural or due to surgical intervention was not recorded. Also at the baseline visit, participants were asked if they had ever been prescribed HRT. Twenty-three participants responded that they were *currently* taking HRT. Due to the small sample size of the latter group, this was combined with the participants who had been prescribed HRT in the past, generating 264 participants in the group with HRT history and 365 participants with no HRT history. APOE4 status was obtained through blood donation, available for all 629 eligible participants.

#### 2.2.2 Co-morbidities and lifestyle variables

Participants were asked if they had ever been diagnosed with cancer, hypertension or diabetes by a general or specialist practitioner. Self-reported endorsements of diagnosis of co-morbidities were chosen for this study based on data availability, power and previous HRT and cognition literature where such comorbidities were indicated as confounders.

Participants were assessed on self-report measures relating to lifestyle. A history of smoking was ascertained through the questions: “Do you smoke?” or “Have you ever smoked?”. For alcohol or drinking behavior, participants were categorized into four groups based on their self-reported frequency of alcohol consumption over the past 12 months: nearly abstinent (no alcohol consumption or drinking once or twice a year); infrequent drinkers (consuming alcohol once or twice a month or once every few months); frequent light-to-moderate drinkers (drinking once or twice a week or three to four times a week); and regular drinkers (drinking five to six times a week or almost daily).

Participants' level of physical activity was assessed based on how frequently participants engaged in 18 different activities classified by intensity: mild (such as light gardening, bowls, light housework, and home repairs), moderate (such as gardening, using an electric lawn mower, car cleaning, moderate-paced walking, dancing, stretching exercises, and heavy housework), and vigorous (such as jogging, swimming, cycling, aerobics or gym activities, tennis, heavy gardening, and manual lawn mowing). A continuous scale was created by multiplying the reported frequency of activity (scored as 0 = once a year or less, 1 = several times a year, 2 = several times a month, 3 = several times a week, and 4 = every day or nearly every day) by the intensity ratio (mild: moderate: vigorous = 1:2:3). This ratio was established according to the metabolic equivalent of task (MET) values recommended in the literature (Aaron et al., 1995).

Self-reported information on the frequency of alcohol consumption over the last 12 months was used to classify participants into four groups: nearly abstinent (not at all in the last 12 months or once or twice a year); infrequent drinkers (once or twice a month or once every couple of months); frequent light-to-moderate drinkers (once or twice a week or three or four times a week); and regular drinkers (five or six times a week or almost every day).

To capture the overall dietary pattern, a total healthy diet score was created. CFAS-Wales assessed the frequency (never, seldom, once a week, 2–4 times a week, 5–6 times a week, or daily) and the daily servings of various foods, including fresh fruit, green leafy vegetables, other vegetables, fatty fish, other fish, wholemeal/brown bread, starch foods, dairy foods, and sugary foods. This analysis specifically focused on the intake frequency of “Mediterranean style” foods, such as fresh fruit, green leafy vegetables, other vegetables, fatty fish, other fish, and wholemeal/brown bread. The frequency was categorized into six levels: never, seldom, once a week, 2–4 times a week, 5–6 times a week, or daily. As per Clare et al. (2017), the total healthy diet score was generated based on these six frequency levels, with scores ranging from 2 (least frequent intake) to 30 (most frequent intake).

#### 2.2.3 Cognition

Baseline cognitive function was assessed using the MMSE (Folstein et al., 1975) for screening (only participants with scores ≥25 were included in the analysis) and The Cambridge Cognitive Examination (CAMCOG, Roth et al., 1986), a standardized assessment tool comprising 67 items designed to evaluate cognitive function across eight domains. These domains include orientation, comprehension, expression, various aspects of memory (such as remote, recent, and learning), attention and calculation, praxis, abstract thinking, and perception. The total possible score ranges from 0 to 107, with lower scores indicating poorer cognitive performance. In this study, both the baseline and follow-up CAMCOG scores were used in these analyses. The change in CAMCOG scores was calculated as the difference between baseline and follow-up CAMCOG scores and were also included in the analyses.

### 2.3 Analytic approach

Multiple linear regression models were employed to investigate the relationship between the independent variables and cognitive outcomes. Initially, a series of linear univariate regression models were fitted to study the association of the CAMCOG follow-up score, with menopausal age, education, history of HRT use, and APOE4 carrier status as predictors. Next, a multivariable linear regression model was fitted including all predictors. Finally, a fully adjusted model that also including an interaction term between HRT use and APOE carriership was also fitted.

Finally, we repeated the same steps considering change in CAMCOG scores between baseline and follow-up as the dependent variable, that is, we fitted a series of univariate models with menopausal age, education, history of HRT use, and APOE4 carrier status as predictors and then fitted a fully adjusted model. Significance of each predictor was assessed using *p*-values associated with the regression coefficients (*p* < 0.05). Results are reported with 95% confidence intervals, and assumptions of normality, linearity, and homoscedasticity were checked for each model via inspection of diagnostic plots. The model fit was evaluated using *R*-squared and adjusted *R*-squared values, and the overall model significance was tested using the *F*-statistic. Finally, Variation inflation factor (VIF) values were examined for issues with multicollinearity. All statistical analyses were conducted using Stata version 17, with a significance level set at *p* < 0.05.

## 3 Results

Sample characteristics and averages for variables of interest are shown in Table 1 for all participants included in this study. Briefly, participants had a mean age of 72.95 years and were ~20 years post menopause (mean age of menopause 49.28 years). Nearly 42% of participants had a history of HRT use and about a quarter (25.25%) were APOE4 carriers.

**Table 1:** Descriptive statistics of analytical sample.

**Variable**	***n***	**Mean or %**	**SD**	**Min**	**Max**
Age at baseline	629	72.95	6.22	65	97
Education (years)	629	12.06	2.65	1	28
Age of menopause	629	49.28	6.17	23	65
HRT use history	264	41.97			
ApoeE4 carrier status	159	25.28			
**Co-morbidities**					
Cancer	107	17.01			
Diabetes	20	3.18			
Hypertension	289	45.95			
**Lifestyle variables**					
Current alcohol	629	2.30	1.08	1	4
History of smoking	629	0.47	0.58	0	2
Total physical activity	629	19.6	13.9	0	88
Current diet	629	18.80	3.80	6	28
**Cognition**					
MMSE baseline	629	28.17	1.55	25	30
CAMCOG baseline	629	91.33	5.38	61	102
CAMCOG follow-up	629	90.83	7.69	12	103
CAMCOG change	629	−0.5	6.24	−78	20

HRT, hormone replacement therapy; MMSE, mini-mental state examination; CAMCOG, Cambridge Cognitive Examination.

Results of the first regression analysis modeling cognitive performance at follow-up showed that the overall model was statistically significant, *F*_(12;615)_ = 13.68, *p* < 0.001, with an *R*^2^ = 0.21, indicating that ~21% of the variance in CAMCOG follow-up scores was explained by the predictors. Results are shown in Table 2. Menopausal age, history of HRT use, alcohol consumption, healthy diet, education in years and age at baseline were significant predictors (*p* < 0.05). A diagnosis of diabetes showed an association that approached significance (*p* = 0.08).

**Table 2:** Results from univariate and fully adjusted regression models fitted to cognitive function at follow-up.

	**Univariate models**			**Fully adjusted models**		
**Variable**	**Coefficient (SE)**	***p*** **-value**	**95% CI**	**Coefficient (SE)**	***p*** **-value**	**95% CI**
Age at menopause	0.10 (0.05)	0.043	(0.03, 0.19)	0.09 (0.05)	**0.04**	(0.01, 0.18)
Education level	0.61 (0.11)	**0.00**	(0.39, 0.83)	0.53 (0.11)	**0.00**	(0.32, 0.73)
HRT use	2.76 (0.61)	**0.00**	(1.56, 3.97)	1.39 (0.59)	**0.02**	(0.24, 2.55)
APOE4 carrier status	−0.03 (0.70)	0.96	(−1.41, 1.35)	−0.29 (0.64)	0.65	(−1.55, 0.96)
Age	−0.42 (0.040	**0.00**	(−0.51, −0.33)	−0.33 (0.05)	**0.00**	(−0.43, −0.23)
Cancer diagnosis	−0.37 (0.81)	0.64	(−1.98, 1.22)	0.29 (0.74)	0.69	(−1.16, 1.74)
Diabetes diagnosis	2.05 (1.35)	0.13	(−0.63, 4.71)	2.78 (1.59)	0.08	(−0.34, 5.89)
Hypertension	−1.7 (0.61)	**0.005**	(−2.92, −0.52)	−0.26 (0.58)	0.66	(−1.39, 0.88)
Alcohol consumption	1.38 (0.27)	**0.00**	(0.83, 1.93)	0.89 (0.36)	**0.00**	0(.37, 1,.41)
Smoking status	1.26 (0.52)	**0.02**	(0.23, 2.28)	0.55 (0.48)	0.25	(−0.39, 1.50)
Physical activity	0.10 (0.02)	**0.00**	(0.06, 0.14)	0.03 (0.02)	0.14	(−0.01, 0.08)
Healthy diet	0.32 (0.08)	**0.00**	(0.17, 0.48)	0.18 (0.08)	**0.02**	(0.03, 0.33)
Constant				107.56 (4.04)	0.00	(99.63, 115.5)

HRT, hormone replacement therapy.

Bold *p*-values indicate significant *p* < 0.05.

Results of the second analysis modeling change overtime, operationalized as the difference between baseline and follow-up CAMCOG scores are shown in Table 3. The overall model was statistically significant, *F*_(12, 615)_ = 2.58, *p* = 0.002, with an *R*^2^ = 0.048, indicating that ~4.8% of the variance in the change in cognitive scores was explained by the predictors. Age at baseline was the only significant predictor in this subsequent model (*p* < 0.05).

**Table 3:** Results from univariate and fully adjusted regression models fitted to cognitive change over time.

	**Univariate models**			**Fully adjusted models**		
**Variable**	**Coefficient (SE)**	***p*** **-value**	**95% CI**	**Coefficient (SE)**	***p*** **-value**	**95% CI**
Age at menopause	0.05 (0.04)	0.21	(−0.03, 0.13)	0.05 (0.04)	0.20	(−0.03, 0.13)
education level	0.15 (0.09)	0.10	(−0.03, 0.33)	0.13 (0.09)	0.17	(−0.06, 0.31)
HRT use	1.19 (0.50)	**0.02**	(0.20, 2.18)	0.65 (0.52)	0.22	(−0.38, 1.68)
APOE4 carrier status	−0.33 (0.57)	0.56	(−1.45, 0.79)	−0.43 (0.57)	0.45	(−1.55, 0.69)
Age	0.05 (0.04)	0.21	(−0.03, 0.13)	−0.14 (0.04)	**0.00**	(−0.23, −0.06)
Cancer diagnosis	−0.36 (0.66)	0.58	(−1.66, 0.94)	−0.08 (0.66)	0.91	(−1.37, 1.22)
Diabetes diagnosis	1.67 (1.09)	0.12	−0.47, 3.85)	1.34 (1.41)	0.34	(−1.43, 4.12)
Hypertension	−0.59 (0.49)	0.23	(−1.57, 0.38)	−0.07 (0.51)	0.90	(−1.07, 0.94)
Alcohol consumption	0.47 (0.23)	**0.04**	(0.02, 0.92)	0.28 (0.24)	0.23	(−0.18, 0.75)
Smoking status	0.42 (0.42)	0.31	(−0.48, 1.26)	0.15 (0.43)	0.73	(−0.69, 0.99)
Physical activity	0.04 (0.02)	**0.02**	(0.007, 0.07)	0.02 (0.02)	0.34	(−0.02, 0.06)
Healthy diet	0.08 (0.06)	0.20	(−0.04, 0.21)	0.03 (0.07)	0.71	(−0.11, 0.16)
Constant				7.88 (3.60)	0.03	(0.81, 14.96)

HRT, hormone replacement therapy.

Bold *p*-values indicate significant *p* < 0.05.

The interaction term of HRT history and APOE carriership did not emerge as statistically significant in either of the two fully adjusted models.

Due to criticisms regarding the sensitivity of the MMSE cut-off threshold of 25 as indicative of cognitive decline, we conducted sensitivity analyses with a revised cut-off threshold of 27 in keeping with previous work (Kukull et al., 1994). The results of this sensitivity analysis did not change the interpretation of our findings for the change in CAMCOG scores over time, but did affect interpretation of the CAMCOG at follow-up model (see Supplementary Table S1), with HRT use and age at menopause no longer contributing to the model with the revised MMSE cutoff score. We further assessed whether any participants transitioned from “normal cognition” to “cognitive impairment” based on MMSE scores at baseline and follow-up. Fifty-five women scoring above 25 on the MMSE at baseline, scored below 25 on the MMSE at the follow-up timepoint. A logistic regression was carried out to assess the relationship between our variables of interest and cognitive impairment. The final log-likelihood of the model was −165.73, and the model yielded a likelihood ratio chi-square (LR χ^2^) statistic of 31.98, *p* = 0.0014, indicating that the overall model was statistically significant. The Pseudo *R*^2^ value was 0.088, suggesting that ~8.8% of the variance in cognitive impairment was explained by the included predictors. Only education level and adherence to a healthy diet were significant predictors (see Table 4).

**Table 4:** Results of fully-adjusted logistic regression model to assess risk for cognitive impairment at follow-up.

**Variable**	**B (SE)**	***z***	***p***	**95% CI**
Age at menopause	−0.01 (0.02)	−0.4	0.67	(−0.06, 0.04)
Education level	−0.22 (0.07)	−2.9	**0.003**	(−0.36, −0.07)
HRT use	−0.45 (0.35)	−1.3	0.19	(−1.13, 0.22)
APOE4 carrier status	0.14 (0.34)	0.42	0.67	(−0.52, 0.81)
Age	0.03 (0.03)	1.19	0.23	(−0.02, 0.08)
Alcohol consumption	−0.25 (0.15)	−1.7	0.09	(−0.54, 0.04)
Smoking status	−0.15 (0.26)	−0.6	0.56	(−0.66, 0.36)
Physical activity	0.00 (0.01)	0.13	0.9	(−0.02, 0.03)
Healthy diet	−0.09 (0.04)	−2.3	**0.02**	(−0.17, −0.01)
Cancer diagnosis	−0.31 (0.42)	−0.7	0.46	(−1.12, 0.51)
Hypertension	0.19 (0.31)	0.61	0.54	(−0.42, 0.80)
Diabetes diagnosis	0.01 (0.45)	0.02	0.98	(−0.88, 0.90)
Constant	−2.24 (2.10)	−1.1	0.29	(−6.36, 1.87)

HRT, hormone replacement therapy.

Bold *p*-values indicate significant *p* < 0.05.

## 4 Discussion

This study explored the complex relationships between HRT use, age at menopause, APOE4 carrier status, lifestyle variables, presence of comorbidities and cognitive outcomes in postmenopausal older-adult women, contributing to the ongoing investigation regarding the efficacy and risks associated with HRT in the context of aging and cognitive decline. In the first analysis which modeled absolute cognitive performance at a specific timepoint, age at menopause, history of HRT, alcohol consumption and healthy diet, along with age and education levels, were significantly associated with cognitive performance. However, the second analysis modeling change in cognitive performance over time revealed that only age remained a significant predictor. The discrepancies in findings between these model approaches may differentiate between predictors that support stable cognitive performance and those that actively contribute to cognitive decline or improvement in older-adult women; these findings will be considered in turn in relation to previous research.

A significant positive association between HRT use and cognitive function, as measured by the CAMCOG scores at follow-up, is consistent with previous observational studies reporting better cognitive performance associated with HRT use (Saleh et al., 2023; Yaffe et al., 1998; Hogervorst et al., 2000); however, the finding that this association did not persist with change over time is in keeping with several RCT studies finding no evidence for HRT to delay cognitive decline in older-adult women (Espeland et al., 2010; Shumaker et al., 2003) as well as an observational study of post-menopausal older-adult women in which HRT history showed no effects on cognitive change over a 3-year follow-up (Wood Alexander et al., 2024). Instead, this shift in significance when change in cognitive scores over time is applied, indicates that while HRT and the other variables of interest, may be associated with cross-sectional cognitive function at a specific time point, they do not necessarily predict the cognitive change on trajectory toward dementia. Nonetheless, the follow-up period of 2 years in this study is relatively restricted and a longer follow-up period with more interim assessments might reveal long-term benefits in rate of cognitive change or decline.

Similarly, age at menopause showed an association with cognitive outcome at follow-up, with earlier menopause associated with poorer cognitive performance at this timepoint. This finding aligns with studies reporting that early menopause increases the risk of Alzheimer's disease (Liao et al., 2023), and suggests that early intervention might mitigate this risk (Coughlan et al., 2023). Again, as the association with menopausal age association did not persist in our model of change in cognitive performance this suggests that either the benefit of later menopausal age does not translate to reduced cognitive decline long-term or the underlying pathophysiological benefits of a longer fertile window could not be indicated by our global cognitive measure. Greater menopausal age may confer a longer fertile window—the period from menarche to menopause—and therefore might represent prolonged exposure to endogenous estrogens providing neuroprotection. Women with longer fertile windows show lower levels of p-tau and t-tau biomarkers relative to those with shorter windows (Lee et al., 2024), substantiating the role of reproductive or sex hormones in mediating AD pathology. Unfortunately, the age of first menses was not available in the current dataset, and thus we could not estimate participant's fertile window in this study. Lee et al. (2024) also considered the duration of HRT use, reporting that longer durations of HRT, particularly when initiated within the critical window of 5 years after menopause, were associated with better cognitive outcomes. Conversely, prolonged use of HRT when started later after menopause did not show the same cognitive benefits and, in some cases, could be associated with increased risks, as found in the (Shumaker et al., 1998) RCT study.

Given proposed caveats of the sensitivity of the MMSE to detect cognitive impairment, we conducted sensitivity analyses including participants with a cut-off score > 27 (Kukull et al., 1994). Under these conditions, HRT use and age at menopause no longer contributed to the model for cognitive performance at follow-up, indicating that the effects of HRT use and age at menopause might be more apparent for maintaining cognitive health in participants with significant impairment (< MMSE 25). The new model might also include participants across a broader range of cognitive abilities, potentially introducing more variability and obscuring the effects of HRT use and age at menopause. Alternatively, these discrepancies might reflect an artifact of the MMSE itself, with it not being sensitive enough to capture subtle cognitive changes in higher-functioning individuals (those scoring closer to 30). The ceiling effect could artificially dampen or obscure true associations in the model, making it appear as if certain factors are less relevant.

In our first model two lifestyle factors, current alcohol consumption and greater adherence to a Mediterranean-like healthy diet, were found to be associated with better cognitive performance. Interestingly, a traditional Mediterranean diet typically includes the moderate consumption of red wine and there may be cumulative benefits of the two components measured in our study. The relationship between alcohol consumption behaviors and later-life cognitive health is controversial, but similar evidence in older-adult women suggests that moderate drinkers (consuming < 15 g or approximately one drink per day) show better cognitive scores than non-drinkers (Stampfer et al., 2005). This finding has been further substantiated in moderate drinkers through systematic review (Ran et al., 2021) and through dose-response analysis for female drinkers, specifically (Brennan et al., 2020). Nonetheless, as the Lancet Commission report (Livingston et al., 2024) suggests there is a lack of definitive evidence that non-drinkers are at increased risk of dementia and that excess risk for non-drinkers may in fact be an artifact of current non-drinkers' abstinence at the time of study collection being a response to previously excessive consumption that was not captured. Alternatively, perhaps these relationships are influenced by sex and the heterogeneity in the literature reflects the lack of sex-specific reporting. The finding that more frequent intake of foods associated with the Mediterranean diet shows a positive association with cognitive performance resonates with several previous works (see Siervo et al., 2021 for review). Indeed, higher adherence to this dietary pattern has been associated with a significantly lower risk of mild cognitive impairment (RR = 0.91, 95% CI = 0.85–0.97) and lower risk of AD (RR = 0.89, 95% CI = 0.84–0.93) through dose-response meta-analysis (García-Casares et al., 2021). Specific to women, the diet's focus on plant-based foods with high degrees of polyphenolic and phytoestrogenic compounds and purported estrogenic properties (Barrea et al., 2021) may support the menopausal transition and possibly offset the influence of cumulating age-related and post-menopausal health conditions, such as cardiovascular and metabolic conditions (Szmidt et al., 2023). Our study did not find any influence of comorbidities, such as cancer, hypertension and diabetes, although the latter did show a trend toward significance (*p* = 0.08) in our first model.

Interestingly, no significant association with APOE4 status and cognitive outcome or change was demonstrated, and there was no evidence of an interaction of HRT and APOE4 upon cognition in this study, in contrast to previous studies of diverse older-adults (Saleh et al., 2023; Gharbi-Meliani et al., 2021). Although, the UK Biobank cohort study (*N* = 111,739) found significant interactions between APOE4 status with the presence of cardiometabolic diseases and older age on cognitive abilities, but these effects did not survive correction for confounders, including diabetes, cardiovascular disease and hypertension (Lyall et al., 2016); variables incorporated into our analyses. Longitudinal evidence from a 20 year follow-up study suggests that APOE4 heterozygote carriers showed poorer cognition only upon age 75 years and older (Gharbi-Meliani et al., 2021). Thus, it is possible that our sample may be, on average, too young to be demonstrating group level effects on cognition. Moreover, our study's sample size limitations could contribute to these non-significant findings. Although APOE4 status was recorded for all participants, the relatively small proportion of APOE4 carriers (~25%) may not have provided sufficient power to detect smaller effect sizes in interaction with other factors, such as HRT use and lifestyle behaviors. Nonetheless, the debate surrounding the influence of APOE4 genotype independently or in interaction with other reproductive (Saleh et al., 2023; Lindseth et al., 2023) or lifestyle and health factors (Dhana et al., 2021; Lyall et al., 2019) is deserving of further investigation to resolve these inconsistencies.

While this study contributes valuable insights to predictors of cognitive performance in older-adult post-menopausal women, there are notable limitations. Its cross-sectional design with a limited follow-up period of 2-years restricts its ability to draw causal conclusions. Further, it relied on self-report of health and lifestyle variables, in some instances reducing these factors to presence or history of comorbidities and health behaviors. More detailed and validated medical and lifestyle information (e.g., duration of illness, duration of smoking, history of alcohol consumption behaviors) might have rendered more nuanced or a different set of results. Similarly, the lack of detailed information on HRT formulations, dosages, as well as proxies for fertility, such as parity and length of fertile window may have compromised interpretations of the results obtained in relation to reproductive and fertility proxies. For example, our study could also not address whether the timing of HRT initiation following the menopause was a critical factor, as the HRT initiation date was not recorded as part of the interview. The timing of HRT initiation relative to menopause onset may indeed be crucial in determining its effects on cognitive outcomes and brain structure. In a recent study, women who initiated HRT closer to the onset of menopause, that is, within 5 years, tended to have better cognitive performance and more favorable brain volume outcomes compared to those who started HRT after a longer delay following menopause (Coughlan et al., 2023). Nonetheless, previous work failed to find evidence that HRT initiation close to menopause shows beneficial effects on cognitive function in later life (Ryan et al., 2009). Surgical and natural menopause could also not be differentiated in the dataset, which might have provided a more refined interpretation of these results and in relation to other findings. Women who have had an oophorectomy before their natural age of menopause have a faster rate of cognitive decline and a significantly higher risk of cognitive impairment and dementia later in life (Bove et al., 2014; Phung et al., 2010; Rocca et al., 2007), risks which are particularly pronounced in women who do not initiate HRT at all or soon after surgery (Bove et al., 2014; Rocca et al., 2014).

Importantly, while our sample comprises participants living in urban and rural Wales, offering some socio-economic and socio-linguistic diversity, it is greatly restricted by its lack of ethno-racial diversity. Ethno-racial variations in the prevalence, risk, presentation, and progression of AD have been documented (Demirovic et al., 2003; Guland et al., 1997; Howell et al., 2017; Kulminski et al., 2020). These disparities are likely the result of intricate interactions between genetic susceptibility and risk factors that are influenced by biological, lifestyle, and socio-cultural differences, which vary across ethno-racial groups (Brothers et al., 2019; Xiong et al., 2020). In relation to female-specific risk factors, both the timing of menopause and the associated symptoms, including psychological and vasomotor changes, have been found to vary among different ethnicities (Avis et al., 2001; Thurston and Joffe, 2011). Similarly, this is an older-adult sample (all were aged 65 years and older at baseline), and diversity in ages and thus stages of the fertile window, as well as variations in parity and experiences with HRT and other hormones, including exogenous estrogens, such as hormonal contraception (see Gregory et al., 2023a for review), may have further delineated the complex interplay of factors that influence cognitive outcomes in later life or potentially explain the heterogeneity observed in cognitive aging and Alzheimer's disease risk within the female population. Like many established cohorts, the selected dataset was not designed with sex differences or female-specific risks for dementia in mind, and thus the ability to comprehensively assess all purported sex-specific risks was limited. Nonetheless, there have been calls and initiatives to improve upon current and future data collection methods in cognitive aging cohorts with an embedded focus on female brain health (Udeh-Momoh and Watermeyer, 2021; Watermeyer et al., 2024; Udeh-Momoh et al., 2024).

Future research will benefit from this concerted effort to highlight female-specific dementia risks. These studies should focus on longitudinal follow-up with higher throughout to better understand the long-term effects of HRT on cognitive outcomes, particularly in relation to APOE4 genotype or other genetic factors or biomarkers. Studies exploring broader physiological responses of HRT and its interaction with female-specific risks and factors as well as lifestyle and health factors will also be crucial in providing a clearer picture of how these factors influence dementia risk.

In conclusion, the study provides some support for the influence of HRT, menopausal age and lifestyle factors on later life cognitive function or performance of older-adult women, but no evidence that these factors influence cognitive function over time. The discrepancy between the two analytic models highlights the importance of considering the choice of outcome when interpreting the effects of various predictors on cognitive health, a possible oversight in previous work leading to mixed findings surrounding the influences of HRT history and menopausal age in female cognitive health, in particular. Overall, the pattern of our findings emphasizes the complexity in delineating the roles of HRT and reproductive histories alongside lifestyle and health variables in cognitive aging in women. Future longitudinal work is needed to further illuminate these complex relationships to optimize tailored strategies that support cognitive health in women across the menopause spectrum.

## Data Availability

Publicly available datasets were analyzed in this study. This data can be found at: https://portal.dementiasplatform.uk/CohortDirectory/Item?fingerPrintID=CFAS%20Wales.
