# Supplementary material for: Hormone replacement therapy, menopausal age and lifestyle variables are associated with better cognitive performance at follow-up but not cognition over time in older-adult women irrespective of APOE4 carrier status and co-morbidities
Source: Front Dement. 2025 Jan 17;3:1496051. doi: 10.3389/frdem.2024.1496051 (PMC11782126; doi:10.3389/frdem.2024.1496051)
Supplement: Supplementary file 1 [file Table_1.docx]

**Supplementary Table 1: Sensitivity analysis with participants MMSE≥ 27 cut-off fully adjusted regression models fitted to cognition at follow-up & cognition over time**

|  | **Cognition at follow-up** | | | **Cognition over time** | | |
| --- | --- | --- | --- | --- | --- | --- |
| **Variable** | **Coefficient (SE)** | **p-value** | **95% CI** | **Coefficient (SE)** | **p-value** | **95% CI** |
| Age at Menopause | 0.03 (0.04) | 0.39 | -0.04, 0.11 | 0.00 (0.04) | 0.93 | -0.07, 0.07 |
| Education Level | 0.55 (0.09) | **0.00** | 0.38, 0.73 | 0.25 (0.08) | **0.00** | 0.09, 0.42 |
| HRT Use | 0.55 (0.49) | 0.26 | -0.41, 1.51 | 0.25 (0.46) | 0.59 | -0.65, 1.15 |
| APOE4 Carrier Status | -0.91 (0.55) | 0.10 | -2.00, 0.17 | -0.44 (0.52) | 0.40 | -1.45, 0.58 |
| Age | -0.32 (0.04) | **0.00** | -0.41, -0.24 | -0.16 (0.04) | **0.00** | -0.24, -0.08 |
| Alcohol Consumption | 1.14 (0.49) | **0.02** | 0.18, 2.09 | 0.05 (0.46) | 0.92 | -0.85, 0.95 |
| Smoking Status | -0.13 (0.41) | 0.75 | -0.94, 0.68 | -0.30 (0.39) | 0.44 | -1.06, 0.46 |
| Physical Activity | 0.02 (0.02) | 0.29 | -0.02, 0.06 | 0.00 (0.02) | 0.74 | -0.03, 0.04 |
| Healthy Diet | 0.06 (0.07) | 0.37 | -0.07, 0.19 | -0.01 (0.06) | 0.89 | -0.13, 0.12 |
| Cancer Diagnosis | -0.65 (0.64) | 0.31 | -1.91, 0.61 | -1.06 (0.60) | **0.08** | -2.24, 0.12 |
| Hypertension | 0.58 (0.49) | 0.24 | -0.39, 1.55 | 0.88 (0.46) | **0.06** | -0.03, 1.79 |
| Diabetes Diagnosis | -0.71 (0.76) | 0.36 | -2.21, 0.79 | -0.19 (0.72) | 0.79 | -1.60, 1.21 |
| Constant | 113.46 (3.46) | 0.00 | 106.66, 120.25 | 10.83 (3.24) | 0.00 | 4.46, 17.20 |

HRT: Hormone Replacement Therapy, bold p-values indicate significant p<0.05
